# Dispensing of Prescribed Medicines in Swiss Community Pharmacies-Observed Counselling Activities

**DOI:** 10.3390/pharmacy7010001

**Published:** 2018-12-21

**Authors:** Karen A. Maes, Jasmine A. Ruppanner, Tamara L. Imfeld-Isenegger, Kurt E. Hersberger, Markus L. Lampert, Fabienne Boeni

**Affiliations:** 1 Pharmaceutical Care Research Group, University of Basel, 4056 Basel, Switzerland; karen.a.maes@gmail.com (K.A.M.); j.ruppanner@gmail.com (J.A.R.); tamara.isenegger@unibas.ch (T.L.I.-I.); kurt.hersberger@unibas.ch (K.E.H.); markus.lampert@unibas.ch (M.L.L.); 2 Institute of Hospital Pharmacy, Solothurner Spitäler, 4600 Olten, Switzerland

**Keywords:** community pharmacy practice, dispensing, counselling, pharmaceutical intervention, pharmaceutical care, observation

## Abstract

**Background:** Patient counselling and addressing drug-related problems are the pharmacist’s key activities to ensure the safe and effective use of medicines. This study aimed to describe the dispensing practice of prescribed medicines in daily community pharmacy practice and to identify factors influencing counselling provision; **Methods:** An observational study was conducted in community pharmacies in Basel, Switzerland. One master student in pharmacy performed non-participatory observations for one day at each of the participating community pharmacies. Patient characteristics, counselling content, additional activities, and pharmaceutical interventions were documented on a structured checklist; **Results:** 556 prescription encounters (PE) in 18 participating community pharmacies were observed (269 first prescriptions; 287 refill prescriptions). Patients were regular customers (n = 523, 94.1%) and 53.8 ± 23.4 years old. Counselling was provided to 367 (66.0%) customers on 2.9 ± 3.1 themes per PE. Factors influencing counselling were dispensing by the pharmacist, new customer, customer who did not refuse counselling, customer with a first prescription, with a prescription resulting in a pharmaceutical intervention, and a prescription filled by carers. During 144 PEs, 203 interventions were documented. Pharmacists proposed few additional activities and performed no cognitive pharmaceutical service; **Conclusions:** Our study quantified counselling and additional services at the dispensing of prescribed medicines and identified influencing factors on counselling provision at the patient, prescription, and pharmacy level.

## 1. Introduction

The Pharmaceutical Care Network Europe defined “Pharmaceutical Care” as “the pharmacist’s contribution to the care of individuals in order to optimize medicines use and improve health outcomes provided” [1]. As part of pharmaceutical care, patient counselling and addressing drug-related problems (DRPs) are the pharmacist’s key activities to ensure the safe and effective use of medicines [2,3]. Dispensing includes all activities between the reception of the patient with a prescription and the distribution of medicines to the patient with the provision of counselling [2]. During dispensing, community pharmacists help the patient to make the best use of prescribed medicines by providing written and oral information, responding to the patients’ needs [4]. Patients have the opportunity to receive counselling and education about their health problems and medicines in several care situations, especially in community pharmacies at the time of dispensing prescribed medicines [5]. Patient counselling about their medicines (e.g., administration, risks and benefits) has been shown to be effective in improving medicine adherence [6,7], and in identifying DRPs [8]. In contrast, insufficient information about medicines can lead to patient non-adherence to drug therapy, and negative health outcomes [5]. 

The joint International Pharmaceutical Federation and World Health Organization (FIP/WHO) guidelines on Good Pharmacy Practice (GPP) describes the pharmacist’s function of dispensing medicines concerning counselling as “providing advice to ensure that the patient receives and understands sufficient written and oral information to derive maximum benefit for the treatment” [3]. Prescription dispensing at the community pharmacy is an important contact point for patient counselling [8]. Patients regularly pick up their prescribed medicines in community pharmacies [9], hence pharmacy staff are usually one of the last healthcare providers who interact with patients prior to medication intake that has the possibility to counsel them [10,11]. The joint FIP/WHO GPP also suggests minimum national standards that should be established for this function.

In Switzerland, the Swiss Association of Pharmacists published standards for pharmaceutical counselling [12]. A service–based remuneration system for community pharmacies has been established since 2001 [13] and some cognitive pharmaceutical services are reimbursed by the health insurance [14,15]. The counselling provided during dispensing of prescribed medicines is remunerated by the ‘drug check’ (fixed fee for checking each dispensed item for dosage, interactions, risk factors, contraindications, misuse and for patient counselling, choice of optimal package size, etc.) and ‘delivery check’ (fixed fee for managing a patient record and checking medication history) [14]. In Switzerland, prescribers can issue refill prescriptions for up to 12 months for patients with ongoing long-term therapies, leaving responsibility for counselling and follow-up of therapy to the pharmacist.

The literature on counselling in community pharmacies described the communication between patient and provider about the medicines use [16,17] and compared counselling practice to guidelines [9,18]. A Swiss study described community staff-patient interactions with a focus on adherence [19]. This study showed that only 6.7% of all patient interactions comprised adherence counselling, and recommended an in-depth analysis of pharmacist-patient interaction. To the authors’ knowledge, the full pattern of the daily community pharmacy practice, with all activities and interventions a prescription triggers at the time of dispensing in a setting with remuneration for prescription validation and counselling, have not been described yet. 

For this reason, the aim of the study was to describe the observed dispensing practice of prescribed medicines at the counter in daily community pharmacy practice, focusing on counselling, pharmaceutical interventions and further activities, and to define factors influencing counselling provision.

## 2. Materials and Methods 

A non-participatory observational study was conducted in community pharmacies in Basel, Switzerland to illustrate the observed dispensing practice of prescribed medicines. The Ethics Committee of Northwest and Central Switzerland approved the study on 25.01.2016 (EKNZ BASEC UBE-req. 16/00011).

### 2.1. Data Collection

Community pharmacies in Basel, Switzerland, were randomly invited for study participation, according to a prior study [19]. One master student in pharmacy observed pharmacy staff-customer interactions for one day at each participating community pharmacy in a non-participatory way during March and April 2016. The observation method was based on ad-hoc manual recording of exchanged information during a pharmacy staff-customer interaction and subsequent transcribing into quantitative information. After a quick briefing about the study, the pharmacy staff were neither actively involved in data collection, nor disturbed in their usual practice. At the dispensing of prescribed medicines, counselling content (information exchanged over the counter between customer and pharmacy staff), patient characteristics (e.g., age), pharmaceutical interventions (e.g., dose adjustment) and additional activities (offered and/or performed further activity/service) were documented on a structured checklist for each prescription encounter (PE). The non-participatory observer stood next to a pharmacy staff member and recorded a PE from the greeting of a customer (patient or carer) filling a prescription in the community pharmacy to closing salutations; where after, the next customer was observed. Customers were not informed about the study to avoid any influence on the counselling activities. Only communication in German was assessed.

The checklist was modified from a previous study [20] and enabled ad hoc coding of nine categories and 61 predefined themes: Patient characteristics (n = 4 themes), provider (pharmacy staff involved, n = 1), prescription (n = 7), counselling (n = 34), intervention (n = 2), physician contact (n = 2), situation (n = 6), and additional activities (n = 5). The category counselling, including 34 counselling themes that were considered as best practice, was derived from the ‘drug check’ of the Swiss service–based remuneration system [13], the literature [9,21,22], the requirement of the Omnibus Budget Reconciliation Act (OBRA, 1990) [23], the recommendations for internal audits of the Swiss Pharmacists’ Association [24], and on expert discussions with five community pharmacists. Each theme was defined in a data dictionary to standardize the observer judgement. The checklist enabled to distinguish between the active and passive (e.g., asking and answering questions, respectively) involvement of the pharmacy staff and the customer during the PEs. After piloting, the checklist was refined and cases were discussed between the observer and two experienced community pharmacists to ensure data quality. An anonymized copy of the prescription and list of medicines from refill prescriptions of every observed patient were additionally collected and used to test the documentation of the observed PEs on consistency and plausibility. Observation time and characteristics of the pharmacies and their staff were recorded separately.

The systematic documentation of the pharmaceutical interventions, performed by pharmacists, were accomplished with aid of the Pharmacists’ Documentation of Intervention in Seamless Care (PharmDISC) system. This classified the pharmaceutical interventions in different categories (problem, type of problem, cause, intervention, person involved, and the outcome of the intervention) [25]. 

At the end of the observation day, a semi-structured interview focusing the pharmacists’ opinion on the counselling, triggers, facilitators, and barriers was conducted with one pharmacist per pharmacy. The results of the interviews are reported separately [26]. 

The main outcome measures were the number and type of themes covered in counselling, the factors influencing counselling provision, and number, frequency, and type of pharmaceutical interventions and additional activities.

### 2.2. Data Analysis

All coded data were quantified and analyzed descriptively using IBM SPSS Statistics for Windows, Version 24 (IBM Corp., Armonk, NY, USA). For the determination of factors influencing counselling provision, counselling theme ratios (sum of each counselling theme counselled divided by the number of medicines dispensed on one prescription) were expressed in percentage. A mean counselling theme ratio of 100% represented the maximum of all 34 possible counselling themes counselled for each dispensed medicine. A single factor variance–analysis, Chi-Square, Spearman, and Mann-Whitney U tests were used to compare variables. A *p*-value < 0.05 was considered statistically significant. 

## 3. Results

Forty-nine community pharmacies were invited and 18 (37%) took part in the study. Reasons for participation refusal were no interest (n = 7), lack of staff resources (n = 4) or time (n = 4), holidays (n = 2), or unknown (n = 14). All pharmacies were located in the urban area of Basel. Thirteen were independent pharmacies (72.2%), while five belonged to a pharmacy chain (27.8%). They were on average open for 10.25 ± 1.5 h per day, and were observed during 8 ± 0.6 h (covering 78.0% of opening hours) per day, per pharmacy. The mean number of working staff per pharmacy at the observation day was 5.8 ± 2.6 (1.7 ± 0.9 pharmacists, 2.8 ± 1.7 pharmacy technicians, 1.0 ± 0.3 apprentices, and 0.2 ± 0.7 pharmacists in training).

During the total observation time of 145.5 h (18 observation days), 571 PEs (mean 31.2 ± 6.4 per pharmacy, range 22–45) were documented. Fifteen PEs had to be excluded because no medicines were dispensed (n = 9, e.g., drug not in stock), spoken language was foreign (n = 3), ordered medicines were picked-up (n = 1), physician-ordered medication (n = 1), or no documentation about the dispensed medicines was available (n = 1). A total of 556 PEs (269 first PEs and 287 refill PEs) constituted the sample for statistical analysis (each PE involved one customer).

Table 1 illustrates the characteristics of the patient, prescription, and provider. The number of medicines on a prescription varied from 1 to 25 (mean 3.2 ± 3.2).

### 3.1. Counselling

**Figure 1:**
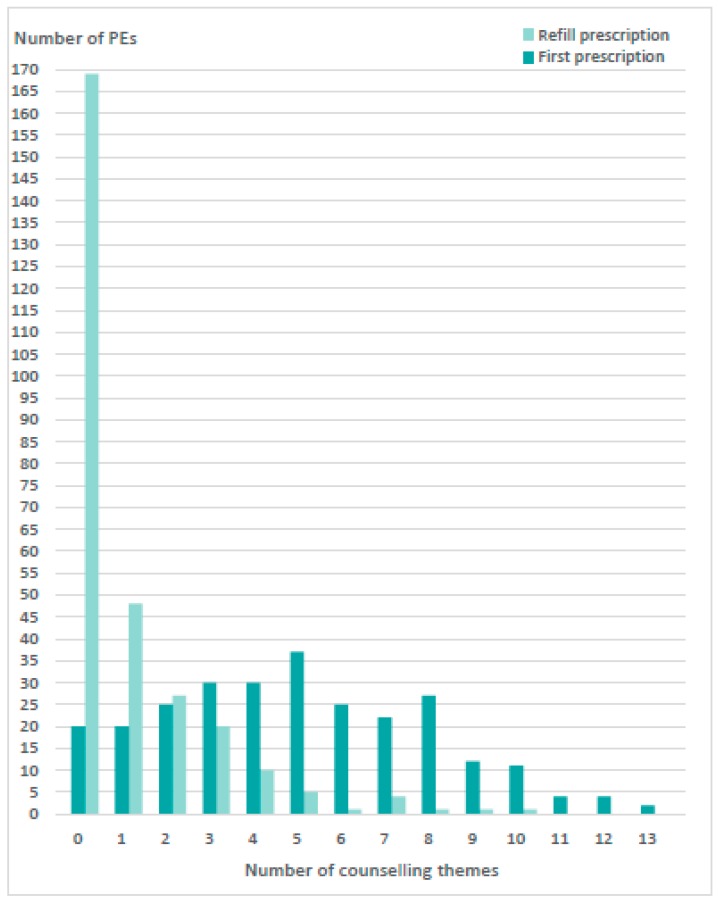
Number of themes counselled by the pharmacy staff per prescription encounter (PE) during first (n = 269) and refill PEs (n = 287).

The PEs lasted on average 4.5 ± 3.0 min (first 5.2 ± 3.1; refill 3.9 ± 2.7, *p* < 0.001), ranging from 1.0 to 23.0 min. In 106 PEs (19.1%), pharmacy staff offered counselling by asking if the patient already knew about their medicines, or if they had any questions regarding the use of medicines (general closed questions that were intended to verify patient need for counselling). During the 556 PEs, counselling was provided to 367 (66.0%) customers, to 249 with first prescriptions and to 118 with refill prescriptions (*p* < 0.001). Of the 367 customers, 68 (12.2%) received counselling on one theme (out of the 34 counselling themes), 52 (9.4%) on two themes, 132 (36.0%) on three to five themes, and 115 (20.7%) on five to thirteen themes (Figure 1). Pharmacy staff did not provide any counselling in 169 refill PEs and in 20 first PEs. On average, customers were counselled on 2.9 ± 3.1 themes per PE (first PEs 4.9 ± 3.0; refill PEs 1.0 ± 1.7, *p* < 0.001). Customers who refused counselling (148 PEs [26.6%]; 51 first PEs vs. 97 refill PEs, *p* < 0.001) were significantly more often approached for counselling at first PEs than refill PEs (3.7 ± 2.9 theme vs. 1.7 ± 1.9, *p* < 0.001).

Table 2 presents the number of the counselling themes and their initiator. Pharmacy staff mainly counselled on administration (at first PEs 465 times and at refill PEs 73 times), dose (188; 46), and use (152; 36) and provided a label (189; 55). Of the 34 counselling themes, 8 were never addressed.

#### 3.1.1. Patient Involvement

The customer was actively involved in 193 (34.7%) of PEs by providing information (n = 149 PEs, 77.2%), asking questions (n = 25, 13.0%), or a combination of both (n = 19, 9.8%). During the first PEs, the customer was more often actively involved than during refill PEs (48.7% vs. 21.6%, *p* < 0.001). 

#### 3.1.2. Factors Influencing Counselling Provision

Table 3 presents factors influencing counselling provision at the patient, prescription and provider level. At patient level, new compared to regular customer received more counselling from the pharmacy staff (11.9% vs. 5.0%, *p* < 0.001) (Table 3). The type of prescription also influenced the rate of counselling. Significantly more counselling was provided with a first compared to a refill prescription (mean theme counselling ratio 9.6% vs. 1.5%, *p* < 0.001), and with prescriptions requiring a pharmaceutical intervention vs. no intervention (7.9% vs. 4.6%, *p* < 0.001). At the provider level, pharmacists provided counselling on significantly more themes per PE than pharmacy technicians (3.5 vs. 2.6 themes, *p* < 0.05), druggists (3.5 vs. 1.9, *p* < 0.05), and apprentices (3.5 vs. 2.3, *p* < 0.05). However, no significant difference between pharmacists and pharmacists in training (3.5 vs. 3.2, *p* = 0.849) or between pharmacists and a combination of a pharmacist and another staff member (3.5 vs. 4.2, *p* = 0.194) was seen.

**Figure 2:**
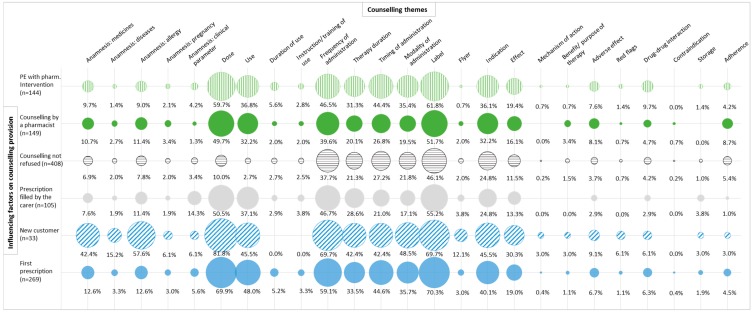
**Patterns of counselling**: Frequency of counselling themes as a function of the factors influencing counselling provision. These factors were selected in terms of significance. The size of the circle represents its frequency with respect to the factors influencing counselling provision. The color and the pattern of the circle help to distinguish the different factors.

The detection of factors influencing counselling provision allowed the illustration of counselling patterns (Figure 2).

### 3.2. Pharmaceutical Interventions

During all 18 observation days, 203 pharmaceutical interventions were documented at 144 PEs (intervention rate 25.9%). Interventions occurred significantly more at first PEs (n = 103) vs. refill PEs (n = 41; *p* < 0.001), with an average per prescription of 1.4 ± 0.7 (range 1–4). Pharmacists’ intervention mainly included drug substitution (n = 89, 43.8%), clarification of information (n = 64, 31.5%), and adjustment of the package size/quantity (n = 39, 19.2%). Table 4 represents the most frequent pharmaceutical interventions. The cause was technical for 180 pharmaceutical interventions (88.7%) and clinical for 23 pharmaceutical interventions (11.3%). Active interaction with the prescriber was necessary in 11 (5.4%) pharmaceutical interventions, whereas the involvement of the patient was observed in 127 (62.6%) pharmaceutical interventions and neither the prescriber nor the patient was involved in 65 (32.0%) pharmaceutical interventions.

The number of pharmaceutical interventions per PE increased with the frequency of counselled themes per PE (correlation r = 0.270, *p* < 0.001) and with the frequency of dispensed medicines per PE (r = 0.236, *p* < 0.001). The number of pharmaceutical interventions per PE did not increase with the age of the patients (r = −0.018, *p* = 0.687) or the work experience of the pharmacy staff (r = 0.032, *p* = 0.470).

### 3.3. Additional Activities

Of all PEs, 10 PEs resulted in a phone call with the physician, five in a referral to the physician, one PE in a consultation in a separate room, and one PE in a refusal of dispensing. The pharmacists reconstituted seven suspensions, and offered three follow-ups. Non-pharmacological counselling (e.g., balanced nutrition) was provided at 11 PEs (11 first, 0 refill, *p* < 0.001).

## 4. Discussion

This observation study allowed depicting the dispensing practice of prescribed medicines at the counter of Swiss community pharmacies and analysing factors influencing counselling provision.

### 4.1. Counselling

Counselling was provided to 66.0% of the customers receiving prescribed medicines, which is slightly more than in a previous observation study (57.3%) performed in Switzerland in 2010 [19]. A review of worldwide counselling practices on prescribed medicines reported counselling rates from 12% to 100%, when observational methods were used [17]. In this study, the customers were counselled on up to 13 out of the 34 predefined counselling themes per PE. However, in real daily practice, 34 different themes cannot be counselled at one PE, as this would overload the patient. Staff members have to decide on priorities during the interaction with the patient. With refill prescriptions, additional themes can be counselled at following visits. Written information in the form of an individualized label to reinforce verbal communication was provided in 43% of the PEs.

A quarter of customers refused counselling and one-third of the customers did not receive any counselling. The study design did not take into account the long-term relationship between the pharmacy staff and the patient as a regular customer, with pre-existing counselling provided at a prior PE, which could lead to PEs without observed counselling. This is in line with what was observed previously: New customers received more counselling than regular customers. Nevertheless, this study depicted how the patient received the counselling at the counter. The findings revealed that pharmacists were involved in direct patient contact at the counter in only a quarter of all PEs. Pharmacists’ activities, such as drug interaction-check and investigating medication history, have often been done in the back office, and are neither visible, nor communicated to the customer. If pharmacy staff were more transparent and better communicated their activities to the customer during dispensing, this could improve trust and collaboration.

The themes of counselling were more product-centered (e.g., dose, administration) than patient-centered (e.g., adherence, therapy benefit), similar to the findings of other studies [17,19,27]. Indeed, the counselling patterns of Figure 2 illustrate the gaps in patient-centered counselling. Especially for patients refilling prescribed medicines, low counselling ratios were observed. Not addressing the patient-centered counselling themes at refill might be interpreted as a missed opportunity to improve patients’ adherence to their drug therapy. It is known that patients often stop taking their newly prescribed medicines in the first months of therapy (medication non-persistence), because of concerns (e.g., adverse effect) and lack of perceived need (e.g., poor understanding of medicines/disease) [28,29]. To address adherence issues, remunerated cognitive pharmaceutical services (e.g., ‘Polymedication check’, ‘Adherence fee’) were introduced in Switzerland since 2010 [14], but during the observation, none of these services were performed.

Pharmacy staff–customer interactions have been observed before and the findings have been similar internationally. Although countries and guidelines adopted pharmaceutical care as one of the key roles for community pharmacist, they are reported to be delivered only in a limited way [21,30,31,32,33]. Counselling rates are usually rather low, and the content is mostly about the medication (product-centered) [16,17,19]. Patient-centered issues are seldom discussed in a pharmacy staff-customer interaction and pharmaceutical care services are not provided to their full potential [32]. Our study confirms these results although in Switzerland, counselling about prescribed medicines and certain pharmaceutical care services are remunerated by the health insurance. To our knowledge, this is the first study observing the daily practice of dispensing prescribed medicines at the community pharmacy under these conditions. Another possible barrier is the non-conformity of roles and expectations between pharmacy staff and customers [34]. Educational interventions have shown success in improving counselling by pharmacists [35].

#### 4.1.1. Patient Involvement

This observational study showed that the pharmacy staff were the main initiators of discussion, confirming the findings of another observational Dutch study, which videotaped their encounters [9]. A systematic review revealed a mainly passive role of the patient in conversations with healthcare providers [36], even though guidelines encourage interactive communication [5]. This is in line with this observational study; customers asked only a few questions, although these questions might give the pharmacy staff the opportunity to tailor information on patients’ needs [4]. Lack of privacy at the counter [37], lack of interest in pharmacy counselling [38,39,40], and patients’ underestimation of pharmacists’ role in healthcare are possible reasons for patients’ barriers in asking questions [27,34,41]. Nevertheless, the patients’ initiative would be important, knowing that the outcome of a dialogue depends on the person who initiates the discussion [42]. Indeed, in patient-centered care, the patient always comes first, and their needs should drive the PE [43]. Therefore, the patient should be encouraged in PEs to be more active in the discussion. Furthermore, the findings revealed that pharmacy staff sometimes offered counselling only by asking general closed questions (e.g., do you know this medicine already?), limiting the counselling provision and the patient involvement, and consequently not taking into account the patients’ needs. It has been shown that the counselling provided to the patients does not fulfil their information needs [44]. A study exploring advice-giving behavior in British community pharmacies reported that the counselling was mostly based on product use, and that the customers wished information about the drug’s effectiveness, while the pharmacists provided information on drug safety. The authors proposed a protocol to guide pharmacy staff, including the customers’ perspective [27]. To meet patient needs, the pharmacists should better listen to the patients’ problems and provide individualized counselling [45].

#### 4.1.2. Factors Influencing Counselling Provision

Counselling was not equally provided, suggesting that pharmacy staff use different levels of counselling at PEs. If extended counselling at each first and refill PE is not possible in daily practice, pharmacy staff should target counselling for specific situations. However, it is important to notice that each PE offers to the pharmacist an opportunity to interact with the patient and hence to detect DRPs and patients’ concerns. The study findings highlight some factors influencing counselling provision at the patient, prescription and provider level. These indicators could help in prioritizing prescriptions needing in-depth counselling.

Patient level

- New customers were more likely to receive counselling from the pharmacy staff than regular customers. The counselling patterns revealed that the pharmacy staff performed more likely an anamnesis (medicine, diseases, and allergy) with the new customers, while the counselling patterns of the other factors influencing counselling provision were comparable (Figure 2). Similarly, to a review [17], the pharmacy staff mainly counselled on administration, dose and use.
- Customers who did not refuse counselling received more counselling. Refusing counselling did not mean that the patient did not receive any counselling, but such refusal is known to be an important barrier for the provision of counselling [19]. Lack of patient interest is a common phenomenon during counselling in community pharmacy [38,39], up to 41–63% patients decline a counselling offer [33,40], leading to low counselling ratios [40].
- Carers who filled a prescription for a patient received more information on the prescribed medicines than the patients themselves. Possibly, the carer was not present at the consultation with the prescriber and did not receive information on the patient’s drug therapy.

Prescription level

- Customers with a first prescription received more counselling than customers with a refill prescription. In a first PE, it is important to ensure that the patient receives the knowledge for using their medicines correctly [19]. Appropriate drug use is ensured by counselling on therapy duration, dosage, and optimal timing of drug intake [46]. At refill PE, pharmacists could suppose that patients with chronic medication were already informed about their use [47]. They could also be regular customers needing less clarification. Previous studies showed that pharmacy staff classified the communication with patients to be more difficult during refill PE than during the first PE [48,49]. It has been shown that patients’ expectations towards counselling are different in first and refill PEs. More interest by patients during a first PE may facilitate more extensive counselling [40]. This is in line with the study findings: During the first PEs, patients showed more interest in counselling than during refill PEs, as two thirds of the counselling refusals were observed during refill PEs.
- Prescriptions that resulted in a pharmaceutical intervention required more counselling than prescriptions without any intervention, because interventions imply to inform the patient about the DRP, and to involve him/her in solving it. Additionally, these prescriptions must involve the pharmacist, who is known to give more counselling than other pharmacy staff member.

Provider level

- Pharmacists provided more counselling to customers than other pharmacy staff members. Other studies reported this factor as well [19,47,50]. A reason could be that pharmacists have a larger knowledge about drug therapy. Counselling should be driven by the patient and the prescription, not by the randomly allocated pharmacy staff member.

Counselling quality can be improved by developing counselling skills through education (e.g., role-play with standardized patients [51]), patient-centered communication (concordance of provided care with patients’ preferences and needs) [52,53], the implementation of established guidelines on Good Pharmacy Practice [3], and continuing evaluation with feedback (e.g., mystery shopper).

### 4.2. Pharmaceutical Interventions

The study findings confirm that the community pharmacists were effective in detecting, preventing, and solving DRPs [4,54]. By intervening during dispensing, pharmacists contributed to the safe, appropriate, and cost-effective use of drugs. Individual judgement and professional knowledge of the pharmacists and collaboration with the patient, carer, or prescriber was needed to respond satisfactorily to the patient needs. The rate of pharmaceutical interventions (25.9%) was comparable to a German study describing DRPs at time of dispensing prescribed medicines, which reported an intervention rate of 18.0% [54].

### 4.3. Additional Activities 

Pharmacy staff proposed only a few additional activities during PE, missing the opportunity to offer additional care and ensure continuity of care and optimize patient therapy and health outcomes. Notably, each refill prescription is an opportunity for the pharmacists to offer follow-up and further cognitive pharmaceutical services. Although such services are remunerated in Switzerland [14], none of the pharmacists proposed a medication review (e.g., ‘polymedication check’) or an adherence aid (e.g., ‘adherence fee’) to the customer during the observation time of 18 days. However, they often performed ‘generic substitution’ for newly prescribed medicines. This limited practice of pharmaceutical care in community pharmacies confirms the results of previous findings [33,50,55] and indicates that the implementation of these cognitive pharmaceutical services is still challenging.

### 4.4. Strengths and Limitations

The approach of the study to describe the dispensing practice of prescribed medicines at the counter was a non-participant observation, which is a useful way to study the quality of services and consistency of care [56]. The full pattern of the real-life situation, with all of the activities and interventions triggered by a prescription at the time of dispensing could be described, which forms a basis to improve these processes. In general, observations allow the description of customers’ behavior and practice in real daily life [57], avoiding consequently the biases of self-report methods [46]. The observation method used in this study was based on ad-hoc note-taking of exchanged information and subsequent transcribing into quantitative information. This demonstrated that observation is a feasible method to provide valuable insight into pharmacies’ activities. The documentation of the observed PEs has been tested for consistency and plausibility. The data was collected in 18 randomly selected pharmacies; however, the study was restricted to one region in Switzerland. The principal limitation was the presence of an observer, which could positively influence the counselling performance of the pharmacy staff by triggering them to be more aware of their way of approaching customers (the Hawthorne effect) [58]. To minimize this effect, the observer became accustomed to the pharmacy staff prior data collection to make them feel comfortable. Moreover, the observation lasted a whole working day, which allowed observation of normal practice over time. Simulated client methods, such as mystery shopping, could minimize observation bias, but present limitations of their own. The extracted information corresponds to a small part (snapshot) of healthcare practice only, and is therefore hard to generalize to other healthcare situations [59]. The observations were not recorded and not reviewed by a second investigator, which might have limited the reliability of the results.

## 5. Conclusions

The observation of the dispensing practice of prescribed medicines at community pharmacies resulted in a picture about processes and activities triggered by a customer with a prescription in an everyday practice setting. We identified factors influencing counselling provision at the patient, prescription and provider level: Dispensing by the pharmacist, the customer with a first prescription, customer with a prescription requiring a pharmaceutical intervention, carer filling the prescription for a patient, new customer, and customer not refusing counselling. Counselling was not evenly provided, indicating that pharmacy staff counsel different customers to different degrees. The themes of counselling were more product-centered than patient-centered. With a more transparent practice and patient-centered counselling, pharmacy staff could improve to address the patients’ needs on medicines information. Pharmacists intervened frequently, however, only a few additional activities and no further services (e.g., adherence support) were offered. Education of pharmacy staff should focus more on patient-centered counselling and the customers should be informed about the role of the pharmacist. Further research will analyze the pharmacists’ opinions gathered within this project. Interventional studies could be used to investigate factors for enhancing pharmacy staff-customer interactions overcoming known barriers.

## Figures and Tables

**Table 1:** Patient, prescription, and provider characteristics.

Prescription Encounter	All	First	Refill
	(n = 556)	(n = 269)	(n = 287)
**Patient**			
Female n (%)	337 (60.6)	162 (60.2)	175 (61.0)
Mean age (years) ± SD	53.8 ± 23.4	45.6 ± 23.9	61.4 ± 20.2
Regular customer n (%)	523 (94.1)	242 (90.0)	281 (97.9)
Carer filled a prescription for a patient n (%)	105 (18.9)	62 (23.0)	43 (15.0)
**Prescription**			
Ambulatory n (%)	468 (84.2)	212 (78.8)	256 (89.2)
Hospital discharge n (%)	88 (15.8)	57 (21.2)	31 (10.8)
Provider of counselling *			
Pharmacist n (%)	149 (26.8)	70 (26.0)	79 (27.5)
Pharmacy technician n (%)	267 (48.0)	124 (46.1)	143 (49.8)
Apprentice n (%)	86 (15.5)	45 (16.7)	41 (14.3)
Pharmacist in training n (%)	13 (2.3)	8 (3.0)	5 (1.7)
Druggist n (%)	8 (1.4)	1 (0.4)	7 (2.4)
Combination of pharmacy staff n (%)	33 (5.9)	21 (7.8)	12 (4.2)

* Definition of the different counselling providers in Switzerland: Apprentice is a pharmacy technician in their 3-year training; pharmacist in training is a student in her/his last year of the master in pharmacy curriculum; druggist accomplished a 4-year apprenticeship.

**Table 2:** Number of counselling themes and their initiators. Bold *p*-values are considered as statistically significant (*p* < 0.05).

Counselling Themes (n = 34)	First Prescription Encounters (n = 269)					Refill Prescription Encounters (n = 287)					*p*–Value (First vs. Refill Prescription Encounters of Themes Counselled by Pharmacy Staff)
	Theme Counselled (Pharmacy Staff)	Theme Discussed (Pharmacy or Customer)	Pharmacy Staff as Initiator	Customer as Initiator	Initiator Not Known	Theme Counselled (Pharmacy Staff)	Theme Discussed (any Person)	Pharmacy Staff as Initiator	Customer as Initiator	Initiator Not Known	
	n (%)	n (%)	n (%)	n (%)	n (%)	n (%)	n (%)	n (%)	n (%)	n (%)	
**Anamnesis (total)**	100 (37.2)	101 (37.5)	99 (98.0)	1 (1.0)	1 (1.0)	8 (2.8)	9 (3.1)	8 (88.9)	1 (11.1)	0 (0)	
1. Medicines	34 (12.6)	35 (13.0)	33 (94.3)	1 (2.9)	1 (2.9)	3 (1.0)	4 (1.4)	3 (75.0)	1 (25.0)	0 (0)	**<0.001**
2. Diseases	9 (3.3)	9 (3.3)	9 (100)	0 (0)	0 (0)	0 (0)	0 (0)	0 (0)	0 (0)	0 (0)	**0.001**
3. Allergy	34 (12.6)	34 (12.6)	34 (100)	0 (0)	0 (0)	3 (1.0)	3 (1.0)	3 (100)	0 (0)	0 (0)	**<0.001**
4. Pregnancy/lactation	8 (3.0)	8 (3.0)	8 (100)	0 (0)	0 (0)	0 (0)	0 (0)	0 (0)	0 (0)	0 (0)	**0.003**
5. Family anamnesis	0 (0)	0 (0)	0 (0)	0 (0)	0 (0)	0 (0)	0 (0)	0 (0)	0 (0)	0 (0)	-
6. Lifestyle	0 (0)	0 (0)	0 (0)	0 (0)	0 (0)	0 (0)	0 (0)	0 (0)	0 (0)	0 (0)	-
7. Clinical parameter	15 (5.6)	15 (5.6)	15 (100)	0 (0)	0 (0)	2 (0.7)	2 (0.7)	2 (100)	0 (0)	0 (0)	**0.001**
8. Dose	188 (69.9)	191 (71.0)	180 (94.2)	8 (4.2)	3 (1.6)	46 (16.0)	50 (17.4)	46 (92.0)	4 (8.0)	0 (0)	**<0.001**
**Drug use (total)**	152 (56.5)	153 (56.9)	143 (93.5)	9 (5.9)	1 (0.6)	36 (12.5)	38 (13.2)	36 (94.7)	2 (5.7)	0 (0)	
9. Use	129 (48.0)	130 (48.3)	121 (93.1)	8 (6.2)	1 (0.8)	34 (11.8)	36 (12.5)	34 (94.4)	2 (5.6)	0 (0)	**<0.001**
10. Duration of use (single application)	14 (5.2)	14 (5.2)	13 (92.9)	1 (7.1)	0 (0)	1 (0.3)	1 (0.3)	1 (100)	0 (0)	0 (0)	**<0.001**
11. Instruction/training of use	9 (3.3)	9 (3.3)	9 (100)	0 (0)	0 (0)	1 (0.3)	1 (0.3)	1 (100)	0 (0)	0 (0)	**0.009**
**Drug administration (total)**	465 (172.9)	475 (176.6)	437 (92.0)	26 (5.5)	13 (2.7)	73 (25.4)	80 (27.9)	69 (86.3)	8 (10.0)	3 (3.7)	
12. Frequency of administration	159 (59.1)	163 (60.6)	154 (94.5)	6 (3.7)	3 (1.8)	34 (11.8)	37 (12.9)	33 (89.2)	3 (8.1)	1 (2.7)	**<0.001**
13. Therapy duration	90 (33.5)	91 (33.8)	85 (93.4)	4 (4.4)	2 (2.2)	13 (4.5)	13 (4.5)	11 (84.6)	1 (7.7)	1 (7.7)	**<0.001**
14. Timing of administration	120 (44.6)	125 (46.5)	111 (88.8)	6 (4.8)	8 (6.4)	20 (7.0)	24 (8.4)	19 (79.2)	4 (16.7)	1 (4.2)	**<0.001**
15. Modality of administration	96 (35.7)	97 (36.1)	87 (89.7)	10 (10.3)	0 (0)	6 (2.1)	6 (2.1)	6 (100)	0 (0)	0 (0)	**<0.001**
**Written information**											
16. Label	189 (70.3)	189 (70.3)	188 (99.5)	1 (0.5)	0 (0)	55 (19.2)	55 (19.2)	55 (100)	0 (0)	0 (0)	**<0.001**
17. Flyer	8 (3.0)	8 (3.0)	8 (100)	0 (0)	0 (0)	0 (0)	0 (0)	0 (0)	0 (0)	0 (0)	**0.003**
18. Schedule	0 (0)	0 (0)	0 (0)	0 (0)	0 (0)	0 (0)	0 (0)	0 (0)	0 (0)	0 (0)	-
19. Document	0 (0)	0 (0)	0 (0)	0 (0)	0 (0)	0 (0)	0 (0)	0 (0)	0 (0)	0 (0)	-
20. Indication	108 (40.1)	111 (41.3)	98 (88.3)	11 (9.9)	2 (1.8)	21 (7.3)	25 (8.7)	20 (80)	5 (20)	0 (0)	**<0.001**
21. Effect	51 (19.0)	52 (19.3)	49 (94.2)	3 (5.8)	0 (0)	7 (2.4)	7 (2.4)	6 (85.7)	1 (14.3)	0 (0)	**<0.001**
22. Mechanism of action	1 (0.4)	1 (0.4)	1 (100)	0 (0)	0 (0)	0 (0)	0 (0)	0 (0)	0 (0)	0 (0)	0.48
23. Benefit/purpose of therapy	3 (1.1)	4 (1.5)	3 (75)	1 (25.0)	0 (0)	8 (2.8)	9 (3.1)	7 (77.8)	2 (22.2)	0 (0)	0.226
24. Adverse effect	18 (6.7)	18 ( 6.7)	16 (88.9)	1 (5.6)	1 (5.6)	6 (2.1)	7 (2.4)	6 (85.7)	1 (14.3)	0 (0)	**0.011**
25. Red flag	3 (1.1)	3 (1.1)	3 (100)	0 (0)	0 (0)	0 (0)	0 (0)	0 (0)	0 (0)	0 (0)	0.11
26. Drug-drug interaction	17 (6.3)	18 (6.7)	13 (72.2)	5 (27.8)	0 (0)	4 (1.4)	4 (1.4)	4 (100)	0 (0)	0 (0)	**0.003**
27. Contraindication	1 (0.4)	1 (0.4)	1 (100)	0 (0)	0 (0)	0 (0)	0 (0)	0 (0)	0 (0)	0 (0)	0.48
**Appropriate action in case of:**											
28. Missed dose	0 (0)	0 (0)	0 (0)	0 (0)	0 (0)	0 (0)	0 (0)	0 (0)	0 (0)	0 (0)	-
29. Underdose	0 (0)	0 (0)	0 (0)	0 (0)	0 (0)	0 (0)	0 (0)	0 (0)	0 (0)	0 (0)	-
30. Overdose	0 (0)	0 (0)	0 (0)	0 (0)	0 (0)	0 (0)	0 (0)	0 (0)	0 (0)	0 (0)	-
31. Storage	5 (1.9)	5 (1.9)	5 (100)	0 (0)	0 (0)	0 (0)	0 (0)	0 (0)	0 (0)	0 (0)	**0.025**
32. Information transfer	0 (0)	0 (0)	0 (0)	0 (0)	0 (0)	0 (0)	0 (0)	0 (0)	0 (0)	0 (0)	-
33. Adherence	12 (4.5)	12 (4.5)	12 (100)	0 (0)	0 (0)	23 (8.0)	23 (8.0)	22 (95.7)	1 (4.3)	0 (0)	0.116
34. Self-/monitoring	0 (0)	0 (0)	0 (0)	0 (0)	0 (0)	1 (0.3)	1 (0.3)	1 (100)	0 (0)	0 (0)	-

**Table 3:** Factors influencing counselling provision. Bold *p*-values are statistically significant (*p* < 0.05).

Variable 1	Mean Ratio of PEs with at Least One Counselling Theme [%] Average ± SD	Variable 2	Mean Counselling Theme Ratio [%] Average ± SD	*p*-value
**Patient**				
Regular customer [n = 523]	5.0 ± 6.1	New customer [n = 33]	11.9 ± 6.3	**<0.001**
Female patient [n = 337]	5.2 ± 6.1	Male patient [n = 219]	5.8 ± 6.5	0.436
Counselling not refused [n = 408]	6.2 ± 6.7	Counselling refused [n = 148]	3.5 ± 4.6	**0.001**
Prescription filled by the patient [n = 451]	5.1 ± 6.2	Prescription filled by the carer [n = 105]	6.7 ± 6.7	**0.026**
**Prescription**				
First prescription [n = 269]	9.6 ± 6.2	Refill prescription [n = 287]	1.5 ± 3.1	**<0.001**
Ambulatory prescription [n = 468]	5.3 ± 6.2	Discharge prescription [n = 83]	6.7 ± 6.7	0.088
Prescription with interventions [n = 144]	7.9 ± 6.6	No intervention [n = 412]	4.6 ± 6.0	**<0.001**
Hand written prescription [n = 247]	7.5 ± 6.7	Printed prescription [n = 117]	7.0 ± 6.3	0.599
All medicines directly dispensed [n = 495]	5.7 ± 6.4	Some medicines picked up later [n = 61]	3.2 ± 4.6	**0.004**
>1 medicine dispensed [n = 290]	5.7 ± 5.9	1 medicine dispensed [n = 266]	5.2 ± 6.7	**0.027**
>1 medicine on prescription [n = 353]	5.0 ± 5.8	1 medicine on prescription [n = 182]	6.5 ± 7.2	0.129
**Provider of counselling**				
Pharmacist [n = 149]	6.3 ± 6.6	Pharmacy technician [n = 267]	5.0 ± 6.1	**0.018**
		Druggist [n = 8]	2.4 ± 6.8	**0.019**
		Apprentice [n = 86]	4.6 ± 5.4	**0.045**
		Combination of a pharmacist and a other staff member [n = 33]	7.6 ± 7.8	0.476
		Pharmacist in training [n = 13]	6.7 ± 5.7	0.651
**Situation**				
Stress factor by waiting customers [n = 89]	6.5 ± 6.6	No waiting customer [n = 467]	5.3 ± 6.2	0.059
Silent environment [n = 500]	5.4 ± 6.4	Loud environment [n = 56]	5.6 ± 5.9	0.582
No disruption during counselling [n = 550]	5.5 ± 6.3	Disruption during counselling [n = 6]	3.9 ± 4.8	0.610
No communication problem [n = 548]	5.4 ± 6.3	Communication problem [n = 8]	6.9 ± 6.5	0.525

**Table 4:** The most frequently observed pharmaceutical interventions, their cause and type of problem (documented with the PharmDISC system).

Intervention	Cause of Intervention	Type of Problem	n (%)
**Total interventions**			**203 (100.0)**
	**Technical**		180 (88.7)
Clarification/addition of information	Incomplete/unclear prescription	Manifest, reactive	55 (27.1)
Substitution (generic)	Financial burden	Manifest, reactive	49 (24.1)
Substitution	Prescribed drug not available	Manifest, reactive	31 (15.3)
Adjustment of package size/quantity	Financial burden	Manifest, reactive	18 (8.9)
Adjustment of package size/quantity	Financial burden	Manifest, reactive	9 (4.4)
	**Clinical**		23 (11.3)
Adjustment of package size/quantity	Concerns about the treatment	Manifest, reactive	3 (1.5)
Substitution	No concordance with guidelines, only suboptimal therapy possible	Potential, preventive	2 (1)
Substitution	Concerns about the treatment	Manifest, reactive	2 (1)
Therapy stopped/no delivery	Drug-drug interaction	Potential, preventive	2 (1)
In-depth counselling of patient	Drug-drug interaction	Potential, preventive	2 (1)
